# Effect of left ventricular ejection fraction Spectrum on 1‐Year mortality in patients with acute ischemic stroke or transient ischemic attack

**DOI:** 10.1111/cns.14285

**Published:** 2023-06-07

**Authors:** Na Wei, Yufei Wei, Ximing Nie, Xiran Liu, Xianglong Xiang, Yuesong Pan, Xia Meng, Liping Liu, Yongjun Wang

**Affiliations:** ^1^ Department of Neurology, Beijing Tiantan Hospital Capital Medical University Beijing China; ^2^ China National Clinical Research Center for Neurological Diseases Beijing China

**Keywords:** cardiac function, ejection fraction, ischemic stroke, outcome, survival

## Abstract

**Aims:**

We aimed to investigate the association of the left ventricular ejection fraction (LVEF) spectrum with 1‐year clinical outcomes in patients with acute ischemic stroke (AIS) or transient ischemic attack (TIA).

**Methods:**

In a prospective registry for the Third China National Stroke Registry (CNSR‐III), AIS or TIA patients with echocardiography records during hospitalization were recruited. All LVEFs were categorized into intervals of 5% in width. The lowest and highest intervals are ≤40% and >70%, respectively. The primary outcome was all‐cause death at 1 year. Cox proportional hazards regression analysis was performed to investigate the association between baseline LVEF and clinical outcomes.

**Results:**

This analysis included a total of 14,053 patients. In total, 418 patients died during 1‐year follow‐up. Overall, LVEF ≤60% was associated with a higher risk of all‐cause death compared to LVEF >60%, independent of demographic and clinical characteristics (aHR 1.29 [95% CI 1.06–1.58]; *p* = 0.01). The cumulative incidence of all‐cause death was significantly different among the eight LVEF groups that survival declined successively with the decrease of LVEF (log‐rank *p* ≤ 0.0001).

**Conclusions:**

Patients with AIS or TIA with decreased LVEF (≤60%) had a lower 1‐year survival rate after onset. LVEF 50%–60% even within the normal range, may still contribute to poor outcomes in AIS or TIA. Comprehensive evaluation of cardiac function after acute ischemic cerebrovascular disease should be strengthened.

## Graphical abstract

LVEF ≤60% was associated with higher risk of all‐cause death compared to LVEF >60%, and the risk of all‐cause death increased gradually with decreasing LVEF levels. LVEF 50%–60% even within the normal range may still contribute to poor outcomes in acute ischemic cerebrovascular disease.

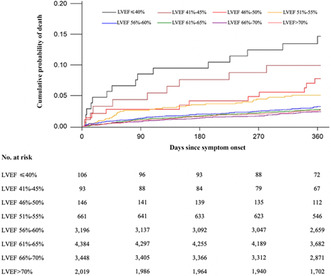

## 1 INTRODUCTION

Left ventricular ejection fraction (LVEF) is the most widely used indicator for quantifying left ventricular systolic function and is associated with cardiovascular outcomes.^1^ Recently, a large sample size cohort study based on community populations from a regional healthcare system demonstrated a U‐shaped relationship between all‐cause death and LVEF. Notably, deviation of LVEF from 60% to 65% was associated with poorer survival regardless of age, sex, or other relevant confounders, which arouse the attention to the people with supra‐normal LVEF.^2^

Although LVEF is routinely reported in patients with acute ischemic cerebrovascular disease during hospitalization, its impact on clinical outcomes has not been adequately investigated. Previous studies have typically investigated LVEF within a specific range (LVEF <40%) or classified it into a crude dichotomization (LVEF ≥50% vs. <50%), mainly in patients with heart failure, with limited sample sizes, or lack of long‐term follow‐up.^3^, ^4^, ^5^, ^6^, ^7^ However, none of these studies have investigated the association of broad LVEF spectrum with survival in patients with acute ischemic cerebrovascular disease in a large‐scale clinical practice data. And whether supra‐normal LVEF leads to increased risk remains unknown in these population.

We hypothesize that data from a large‐scale clinical registry study will provide new insights into the impact of LVEF spectrum on clinical outcomes in patients with acute ischemic cerebrovascular disease. In this large, nationwide, multicenter, long‐term follow‐up prospective cohort registry for acute ischemic cerebrovascular disease, we aimed to investigate the relationship between the LVEF spectrum and clinical outcomes and to explore whether supra‐normal LVEF increased the risk of poor outcomes.

## 2 METHODS

### 2.1 Study design and participants

Participants were recruited from CNSR‐III (Third China National Stroke Registry).^8^ The CNSR‐III is a nationwide prospective registry that enrolled patients with acute ischemic stroke (AIS) or transient ischemic attack (TIA) performed in 201 hospitals across China between August 2015 and March 2018.^8^ Participants were consecutively enrolled if meeting the following criteria: (1) >18 years old; (2) diagnosis of AIS or TIA within 7 days; (3) informed consent from participant or legally authorized representative. The study design and methods of the CNSR‐III have been reported previously.^8^ Patients in the CNSR‐III with echocardiography records during hospitalization were recruited to the current analysis. Those with missing data for LVEF were excluded from this analysis.

### 2.2 Standard protocol approvals, registrations and patient consents

The study protocol was evaluated and approved by the ethics committee of Beijing Tiantan Hospital and each participating site. All patients or their legal representatives provided written informed consent to participate.

### 2.3 Data collection

All data were collected by trained investigators and monitored by an independent contract research organization throughout the study period. Baseline data included patient demographics, risk factors (ischemic stroke, hypertension, diabetes mellitus, dyslipidemia, and chronic kidney disease), smoking status, medical history of heart disease (coronary heart disease, myocardial infarction, atrial fibrillation, valvular heart defect, dilated cardiomyopathy, and heart failure), National Institutes of Health Stroke Scale (NIHSS) score at admission, laboratory test, etiological classification, echocardiography parameters, and medication at discharge. The etiological classification was conducted by the TOAST (Trial of Org 10,172 in Acute Stroke Treatment) criteria.^9^

LVEF estimation was extracted from transthoracic echocardiography results performed during hospitalization. All LVEFs were categorized into intervals 5% in width and inclusive of the higher threshold. The lowest and highest intervals were ≤40% and >70%, respectively. Therefore, we classified patients into eight categories according to the degree of LVEF. A common LVEF of 61%–65% was established as the reference group for all mortality comparisons.^2^ Other echocardiography parameters including local ventricular wall motion abnormalities, septal thickness, posterior wall thickness, and left ventricular end diastolic diameter were also obtained.

### 2.4 Outcomes measurement

Each patient was followed up according to the study protocol by trained research coordinators who were blinded to the baseline clinical information. The primary outcome was all‐cause death at 1 year defined as death from any cause and confirmed by a death certification from the attended hospital or the local civil registry. And the secondary outcomes included stroke recurrence and functional dependence at 1 year. Stroke recurrence was defined as new ischemic stroke and recurrent hemorrhagic stroke (intracerebral hemorrhage and subarachnoid hemorrhage). Functional dependence was defined as modified Rankin Scale (mRS) of 3–5.

### 2.5 Statistical analysis

Continuous variables were presented as mean ± SD or medians (interquartile range). Categorical variables were presented as numbers (percentages). Student's *t* tests or Mann–Whitney *U* tests were used for the comparison of continuous variables. *χ* ^2^ tests or Fisher's exact tests were used for the comparison of categorical variables. Cox proportional hazards regression was performed to model time‐to‐death and time‐to‐stoke recurrence according to LVEF levels, and the results are reported as hazard ratios (HR) with 95% confidence intervals (CI). Multivariate logistic regression was used to analyze the independent associations between LVEF intervals and the functional dependence, and the results are reported as odds ratios (OR) with their 95% CI. In the multivariable analyses, variables including age, sex, height, body mass index, smoking, history of hypertension, history of diabetes, using statins at discharge, using antiplatelets at discharge, using anticoagulants at discharge, using antihypertensives at discharge were adjusted in model 1. In model 2, baseline NIHSS score, white blood cells were further added, to adjust for its potential impact on outcomes. In addition, survival analysis was performed by the Kaplan–Meier method. Survival curves were compared between each LVEF level using log‐rank analysis. All statistical analyses were performed using SAS software v 9.4 (SAS Institute Inc). A two‐tailed value of *p* < 0.05 was considered statistically significant.

## 3 RESULTS

### 3.1 Baseline characteristics

**FIGURE 1:**
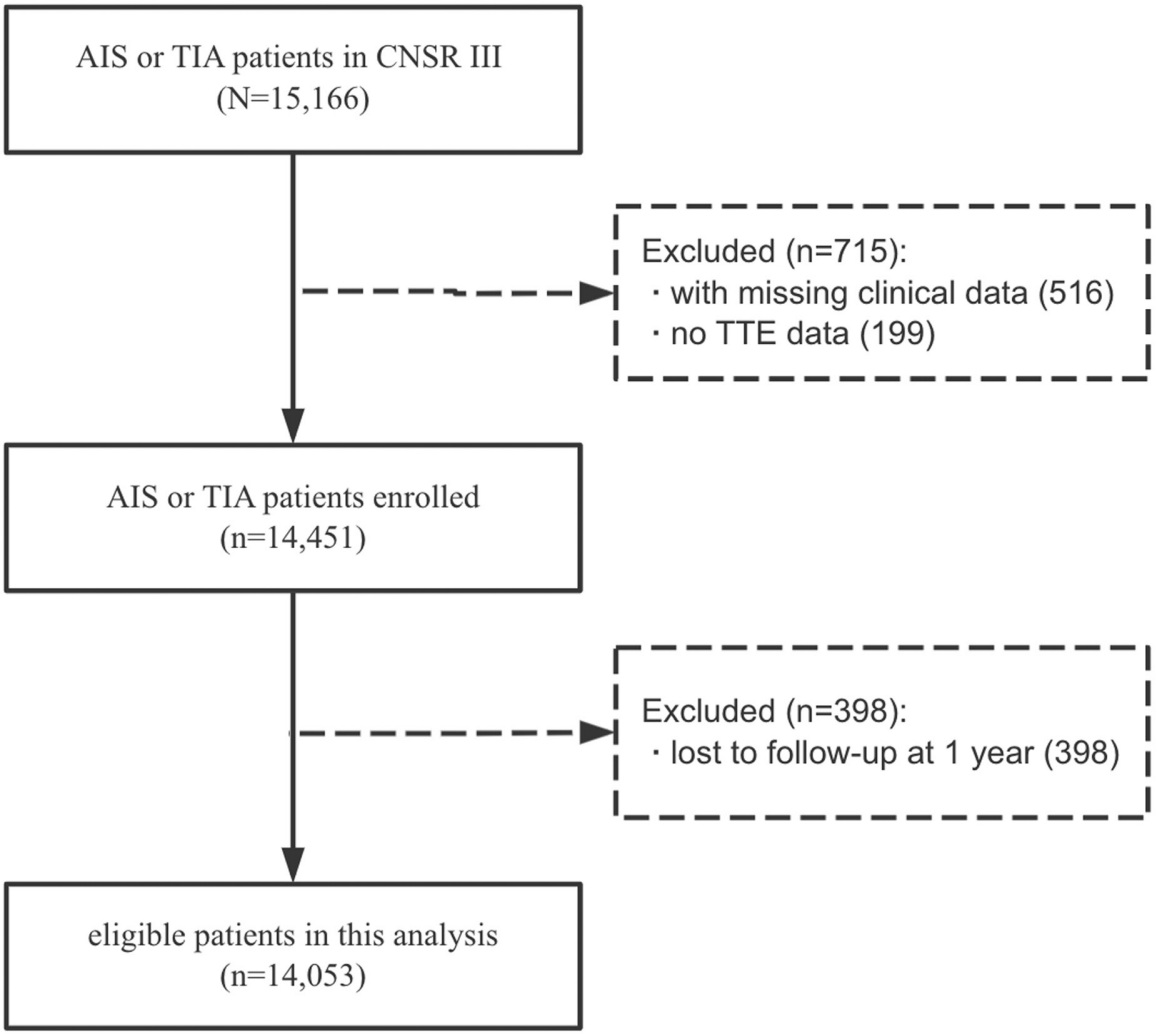
Flowchart of study population selection. AIS, acute ischemic stroke; CNSR‐III, Third China National Stroke Registry; TIA, transient ischemic attack; TTE, Transthoracic echocardiography.

Among the 15,166 patients in the CNSR‐III, a total of 14,451 AIS or TIA patients were recruited in this study by excluding 516 patients with missing clinical data and 199 patients with no transthoracic echocardiography results. After exclusion of 398 patients with missing 1‐year follow‐up, a total of 14,053 patients were finally included in this analysis (Figure 1). The mean age of the study population was 62.2 ± 11.2 years, and 68.2% of all the included patients were male. There was a total of 4202 (29.9%) patients with LVEF≤60%. The demographics and clinical characteristics of included patients are shown among LVEF groups divided by 5% intervals, with the lowest and highest intervals being ≤40% and >70%, respectively (Table 1). Compared with higher values of LVEF groups, patients with lower LVEF were older and more likely to be male; were more likely to have higher height, hematocrit, WBC, C‐reactive protein, blood urea nitrogen, and creatinine level. Lower LVEF patients were more likely to have chronic kidney disease, coronary heart disease, myocardial infarction, atrial fibrillation, valvular heart defect, dilated cardiomyopathy, heart failure, and less likely to have a history of hypertension. Furthermore, patients with lower LVEF had a higher proportion of cardiogenic embolism in TOAST classification, a more severe neurologic deficit on admission, and a higher percentage of receiving anticoagulant agents at discharge, but a lower percentage of receiving antiplatelet agents and statins agents.

**TABLE 1:** Baseline characteristics of patients stratified by each LVEF intervals.

Characteristics	LVEF ≤40% (*n* = 106)	LVEF 41%–45% (*n* = 93)	LVEF 46%–50% (*n* = 146)	LVEF 51%–55% (*n* = 661)	LVEF 56%–60% (*n* = 3196)	LVEF 61%–65% (*n* = 4384)	LVEF 66%–70% (*n* = 3448)	LVEF >70% (*n* = 2019)	*p* Value
Age, years, mean ± SD	64.02 ± 11.17	66.69 ± 9.57	65.58 ± 11.51	64.92 ± 11.18	62.83 ± 11.35	61.83 ± 11.26	61.53 ± 11.10	61.95 ± 11.11	<0.001*
Male, *n* (%)	83 (78.30)	77 (82.80)	117 (80.14)	444 (67.17)	2232 (69.17)	2985 (68.09)	2342 (67.92)	1305 (64.64)	<0.001*
Height, cm, mean ± SD	167.80 ± 8.06	169.43 ± 7.23	168.34 ± 6.68	167.10 ± 7.44	167.39 ± 7.47	166.88 ± 7.42	166.72 ± 7.40	165.94 ± 7.61	<0.001*
BMI, kg/m^2^, mean ± SD	24.93 ± 3.60	24.31 ± 3.95	24.67 ± 3.48	24.77 ± 3.71	24.87 ± 4.21	24.75 ± 3.28	24.67 ± 3.29	24.48 ± 3.36	0.05
Risk factors, *n* (%)									
Previous ischemic stroke	15 (14.15)	26 (27.96)	32 (21.92)	152 (23.00)	682 (21.34)	944 (21.53)	673 (19.52)	388 (19.22)	0.02*
Hypertension	58 (54.72)	53 (56.99)	84 (57.53)	424 (64.15)	1999 (62.55)	2751 (62.75)	2123 (61.57)	1308 (64.78)	0.10
Diabetes mellitus	25 (23.58)	31 (33.33)	32 (21.92)	155 (23.45)	732 (22.90)	1047 (23.88)	767 (22.24)	440 (21.79)	0.16
Dyslipidemia	10 (9.43)	9 (9.68)	8 (5.48)	44 (6.66)	253 (7.92)	352 (8.05)	296 (8.58)	144 (7.13)	0.40
Chronic kidney disease	6 (5.66)	2 (2.15)	1 (0.68)	3 (0.45)	19 (0.59)	32 (0.73)	34 (0.99)	19 (0.94)	<0.001*
Smoking	30 (28.30)	27 (29.03)	42 (28.77)	187 (28.29)	987 (30.88)	1395 (31.82)	1127 (32.69)	612 (30.31)	0.28
Medical history of heart disease, *n* (%)									
Coronary heart disease	40 (37.74)	37 (39.78)	39 (26.71)	108 (16.34)	358 (11.20)	457 (10.42)	314 (9.11)	147 (7.28)	<0.001*
Myocardial infarction	19 (17.92)	15 (16.13)	15 (10.27)	23 (3.48)	72 (2.25)	71 (1.62)	34 (0.99)	17 (0.84)	<0.001*
Atrial fibrillation	36 (33.96)	36 (38.71)	44 (30.14)	107 (16.19)	269 (8.42)	216 (4.93)	137 (3.97)	97 (4.80)	<0.001*
Valvular heart defect	3 (2.83)	2 (2.15)	1 (0.68)	4 (0.61)	11 (0.34)	19 (0.43)	14 (0.41)	5 (0.25)	0.001*
Dilated cardiomyopathy	5 (4.72)	3 (3.23)	0 (0.00)	0 (0.00)	5 (0.16)	1 (0.02)	3 (0.09)	1 (0.05)	<0.001*
Heart failure	11 (10.38)	13 (13.98)	3 (2.05)	12 (1.82)	21 (0.66)	13 (0.30)	11 (0.32)	6 (0.30)	<0.001*
Baseline NIHSS score, median (IQR)	5 (2–10)	4 (2–8)	4 (2–8)	4 (2–7)	3 (1–6)	3 (1–6)	3 (1–5)	3 (1–5)	<0.001*
Laboratory test, median (IQR)									
Hematocrit, %	42.20 (38.60–45.80)	40.70 (35.95–42.90)	41.70 (38.30–44.80)	41.50 (38.10–44.90)	41.00 (36.90–44.50)	41.00 (36.60–44.40)	41.30 (36.90–44.60)	41.00 (36.30–44.20)	<0.001*
WBC, 10^3^/mL	7.47 (6.03–9.12)	7.26 (5.80–8.61)	7.43 (5.99–8.77)	6.88 (5.65–8.50)	6.95 (5.70–8.43)	6.94 (5.73–8.45)	6.87 (5.71–8.36)	6.80 (5.68–8.23)	0.02*
C‐reactive protein, mg/L	3.58 (1.79–10.39)	3.66 (0.96–10.06)	3.57 (1.27–8.50)	1.91 (0.87–4.83)	1.87 (0.87–5.18)	1.73 (0.81–4.45)	1.65 (0.79–4.21)	1.41 (0.71–4.08)	<0.001*
Total cholesterol, mmoL/L	3.72 (3.18–4.76)	4.00 (3.29–4.59)	4.00 (3.32–4.64)	4.06 (3.39–4.83)	4.00 (3.33–4.77)	3.95 (3.31–4.70)	3.97 (3.31–4.70)	3.91 (3.25–4.66)	0.09
Triglyceride, mmoL/L	1.19 (0.93–1.55)	1.28 (0.99–1.70)	1.28 (1.03–1.57)	1.34 (1.01–1.84)	1.35 (1.02–1.84)	1.38 (1.04–1.89)	1.41 (1.04–1.93)	1.36 (1.01–1.88)	0.002*
HDL‐C, mmoL/L	0.97 (0.80–1.13)	0.95 (0.73–1.17)	0.88 (0.76–1.10)	0.96 (0.81–1.16)	0.95 (0.78–1.13)	0.92 (0.78–1.10)	0.93 (0.76–1.11)	0.94 (0.77–1.14)	0.005*
LDL‐C, mmoL/L	2.31 (1.74–3.05)	2.34 (1.91–2.97)	2.37 (1.91–2.96)	2.38 (1.81–3.08)	2.36 (1.75–3.03)	2.28 (1.68–2.94)	2.32 (1.72–2.97)	2.24 (1.70–2.94)	0.03*
Fasting blood glucose, mmoL/L	5.46 (4.90–7.63)	5.77 (5.00–7.97)	5.34 (4.73–6.58)	5.66 (4.91–7.45)	5.52 (4.90–6.97)	5.52 (4.90–6.91)	5.53 (4.90–6.75)	5.43 (4.84–6.65)	0.07
Glycated Hemoglobin, %	6.00 (5.50–7.20)	6.20 (5.70–7.35)	5.95 (5.50–7.00)	5.90 (5.50–7.50)	6.00 (5.50–7.00)	5.90 (5.43–6.90)	5.90 (5.50–6.90)	5.90 (5.50–6.87)	0.64
Blood urea nitrogen, mmoL/L	6.14 (4.88–7.15)	5.12 (4.57–6.50)	5.26 (4.60–6.84)	5.20 (4.13–6.47)	5.05 (4.10–6.16)	4.90 (4.01–6.00)	4.84 (4.05–5.90)	4.90 (4.00–5.90)	<0.001*
Creatinine, μmoL/L	76.00 (63.00–92.00)	77.00 (68.00–88.00)	76.00 (63.00–93.00)	71.00 (60.00–84.00)	70.00 (59.00–83.00)	69.00 (58.00–81.00)	69.00 (59.00–80.00)	69.00 (58.00–80.00)	<0.001*
TOAST classification, *n* (%)									
Large artery disease	23 (21.70)	19 (20.43)	40 (27.40)	169 (25.57)	812 (25.41)	1131 (25.80)	891 (25.84)	507 (25.11)	<0.001*
Cardioembolism	31 (29.25)	23 (24.73)	28 (19.18)	73 (11.04)	245 (7.67)	212 (4.84)	152 (4.41)	94 (4.66)	
Small vessel occlusion	3 (2.83)	12 (12.90)	18 (12.33)	126 (19.06)	639 (19.99)	939 (21.42)	793 (23.00)	451 (22.34)	
Other determined	1 (0.94)	0 (0.00)	1 (0.68)	3 (0.45)	30 (0.94)	63 (1.44)	41 (1.19)	40 (1.49)	
Undetermined	48 (45.28)	39 (41.94)	59 (40.41)	290 (43.87)	1470 (45.99)	2039 (46.51)	1571 (45.56)	937 (46.41)	
Echocardiography									
Local ventricular wall motion abnormalities, *n* (%)	48 (45.28)	45 (48.39)	50 (34.25)	90 (13.62)	167 (5.23)	111 (2.53)	74 (2.15)	63 (3.12)	<0.001*
Septal thickness (mm), mean ± SD	9.92 ± 2.33	10.34 ± 1.83	10.59 ± 1.92	10.37 ± 1.92	10.26 ± 1.70	10.08 ± 1.61	9.93 ± 1.68	10.02 ± 1.81	<0.001*
Posterior wall thickness (mm), mean ± SD	9.28 ± 1.58	9.65 ± 1.37	9.92 ± 1.78	9.91 ± 1.58	9.79 ± 1.52	9.64 ± 1.78	9.58 ± 1.44	9.64 ± 1.37	<0.001*
Left ventricular end diastolic diameter (mm), mean ± SD	58.82 ± 10.51	56.11 ± 7.32	52.05 ± 6.53	48.92 ± 5.60	47.40 ± 4.51	46.73 ± 4.33	46.36 ± 4.25	46.14 ± 4.41	<0.001*
Medication at discharge, *n* (%)									
Statins	87 (82.08)	74 (79.57)	131 (89.73)	609 (92.13)	2869 (89.77)	4015 (91.58)	3181 (92.26)	1886 (93.41)	<0.001*
Antiplatelets	77 (72.64)	66 (70.97)	117 (80.14)	576 (87.14)	2833 (88.64)	4047 (92.31)	3182 (92.29)	1866 (92.42)	<0.001*
Anticoagulants	13 (12.26)	12 (12.90)	18 (12.33)	49 (7.41)	106 (3.32)	82 (1.87)	81 (2.35)	59 (2.92)	<0.001*
Antihypertensives	63 (59.43)	43 (46.24)	63 (43.15)	339 (51.290	1561 (48.84)	2148 (49.00)	1665 (48.29)	1066 (52.80)	0.007*

* *p* < 0.05.

Abbreviations: BMI, body mass index; HDL‐C, high‐density lipoprotein cholesterol; IQR, interquartile range; LDL‐C, low‐density lipoprotein cholesterol; LVEF, left ventricular ejection fraction; NIHSS, National Institutes of Health Stroke Scale; SD, standard deviation; TOAST, Trial of Org 10,172 in Acute Stroke Treatment; WBC, white blood cells.

### 3.2 Clinical outcomes

**FIGURE 2:**
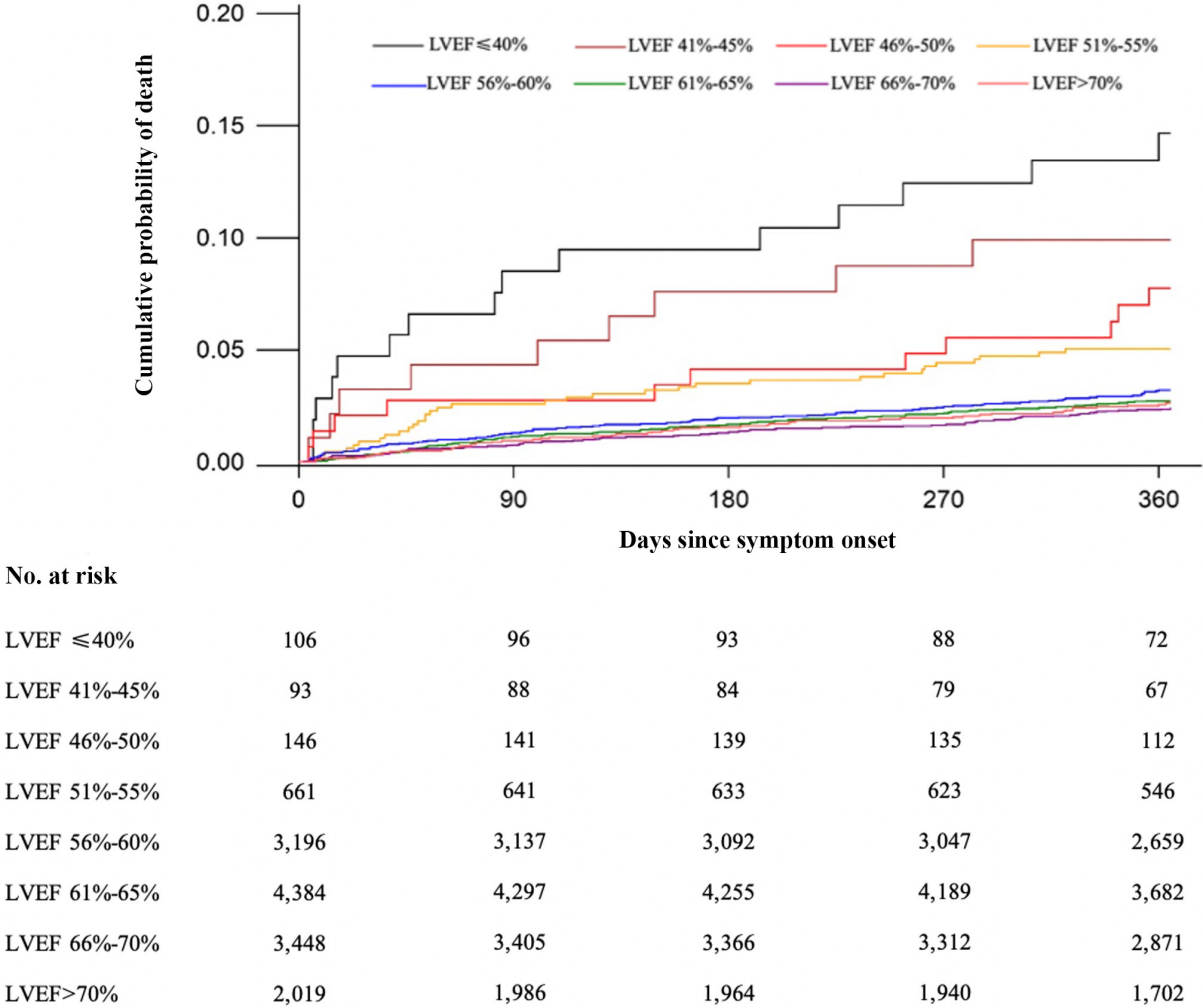
Kaplan–Meier curves of LVEF level on all‐cause death. LVEF, left ventricular ejection function.

**FIGURE 3:**
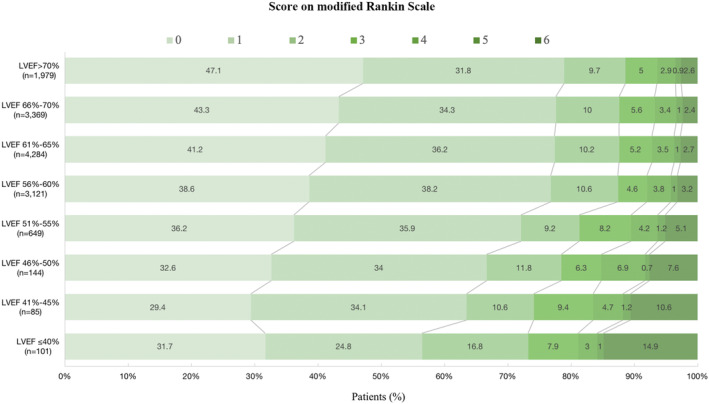
Distribution of the modified Rankin Scale scores at 1 year according to LVEF levels. LVEF, left ventricular ejection function.

The associations of LVEF levels with clinical outcomes at 1 year are shown in Table 2. LVEF **≤**60% was associated with higher risk of all‐cause death compared to LVEF >60% (aHR 1.22 [95% CI 1.01–1.49]; *p* = 0.04), independent of demographic and clinical characteristics (Table 2, model 1). The significant associations of LVEF **≤**60% (aHR 1.29 [95% CI 1.06–1.58]; *p* = 0.01) with the primary outcome remained after further adjusting for variables above and baseline NIHSS score and WBC (Table 2, model 2). Among patients with LVEF ≤60%, a total of 15 (14.15%), 9 (9.68%), 11 (7.53%), 33 (4.99%), and 100 (3.13%) patients died in LVEF ≤40%, 41%–45%, 46%–50%, 51%–55%, and 56%–60% group, respectively. The risk of all‐cause death was reduced as the LVEF levels increased successively in patients with LVEF ≤60% (LVEF ≤40%: adjusted HR 4.27 [95% CI 2.47–7.36]; LVEF 41%–45%: adjusted HR 2.44 [95% CI 1.23–4.84]; LVEF 46%–50% group: adjusted HR 1.77 [95% CI 0.92–3.40]; LVEF 51%–55%: adjusted HR 1.49 [95% CI 1.01–2.20]); LVEF 56%–60% group (adjusted HR 1.01 [95% CI 0.77–1.33]) (Table 2, model 2). The mean survival times of each subgroup were presented based on the Kaplan–Meier estimates. The cumulative incidence of all‐cause death was significantly different among the eight LVEF groups that survival declined successively with the decrease of LVEF (log‐rank P ≤ 0.0001; Figure 2). No significant associations were identified in multivariate logistic regression analyses of secondary outcomes (Table 2, model 2). The distribution of mRS at 1 year according to LVEF levels is shown in Figure 3. A significant shift was observed toward poor functional outcomes in patients with LVEF ≤60% (Figure 3).

**TABLE 2:** Associations of LVEF spectrum with clinical outcomes at 1 Year.

Outcomes	LVEF (%)	*N*	Events, *n* (%)	Crude HR/OR (95% CI)	*p* Value	Model 1 adjusted HR/OR (95% CI)^a^	*p* Value	Model 2 adjusted HR/OR (95% CI)^b^	*p* Value
Primary outcome									
All‐cause death	≤60^c^	4202	168 (39.48)	1.59 (1.31–1.93)	<0.001	1.22 (1.01–1.49)	0.04	1.29 (1.06–1.58)	0.01
	0–40	106	15 (14.15)	5.83 (3.41–9.98)	<0.001	5.00 (2.91–8.60)	<0.001	4.27 (2.47–7.36)	<0.001
	41–45	93	9 (9.68)	3.88 (1.97–7.65)	<0.001	2.62 (1.32–5.18)	0.006	2.44 (1.23–4.84)	0.01
	46–50	146	11 (7.53)	2.89 (1.56–5.36)	<0.001	2.04 (1.09–3.80)	0.03	1.77 (0.92–3.40)	0.08
	51–55	661	33 (4.99)	1.89 (1.28–2.78)	0.001	1.54 (1.05–2.28)	0.03	1.49 (1.01–2.20)	0.04
	56–60	3196	100 (3.13)	1.17 (0.90–1.53)	0.24	1.03 (0.79–1.35)	0.82	1.01 (0.77–1.33)	0.92
	61–65	4384	117 (2.67)	Ref.		Ref.		Ref.	
	66–70	3448	81 (2.35)	0.88 (0.66–1.16)	0.36	0.90 (0.68–1.20)	0.47	0.91 (0.69–1.22)	0.54
	>70	2019	52 (2.58)	0.96 (0.69–1.33)	0.81	0.94 (0.68–1.31)	0.73	0.98 (0.70–1.36)	0.89
Secondary outcome									
Stroke recurrence	0–40	106	16 (15.09)	1.75 (1.06–2.89)	0.03	1.58 (0.95–2.60)	0.08	1.51 (0.91–2.49)	0.11
	41–45	93	7 (7.53)	0.82 (0.39–1.72)	0.59	0.70 (0.33–1.48)	0.35	0.68 (0.32–1.43)	0.31
	46–50	146	17 (11.64)	1.26 (0.78–2.05)	0.34	1.19 (0.73–1.94)	0.48	1.09 (0.66–1.79)	0.75
	51–55	661	56 (8.47)	0.90 (0.68–1.19)	0.47	0.86 (0.65–1.14)	0.29	0.82 (0.62–1.09)	0.17
	56–60	3196	353 (11.05)	1.18 (1.03–1.36)	0.02	1.15 (1.00–1.32)	0.06	1.14 (0.99–1.32)	0.07
	61–65	4384	411 (9.38)	Ref.		Ref.		Ref.	
	66–70	3448	307 (8.90)	0.94 (0.81–1.09)	0.43	0.95 (0.82–1.10)	0.52	0.96 (0.83–1.11)	0.57
	>70	2019	184 (9.11)	0.96 (0.81–1.15)	0.68	0.97 (0.82–1.16)	0.76	0.98 (0.83–1.17)	0.85
mRS 3–5	0–40	86	12 (13.95)	1.47 (0.79–2.74)	0.22	1.31 (0.70–2.46)	0.4	1.17 (0.60–2.25)	0.65
	41–45	76	13 (17.11)	1.88 (1.02–3.44)	0.04	1.64 (0.89–3.04)	0.11	1.56 (0.82–2.96)	0.17
	46–50	133	20 (15.04)	1.61 (0.99–2.62)	0.06	1.40 (0.85–2.31)	0.18	1.33 (0.80–2.23)	0.27
	51–55	616	88 (14.29)	1.51 (1.18–1.94)	0.001	1.35 (1.05–1.74)	0.02	1.24 (0.96–1.62)	0.10
	56–60	3021	293 (9.70)	0.98 (0.83–1.14)	0.77	0.93 (0.79–1.09)	0.39	0.93 (0.79–1.10)	0.42
	61–65	4167	413 (9.91)	Ref.		Ref.		Ref.	
	66–70	3288	336 (10.22)	1.03 (0.89–1.20)	0.66	1.06 (0.91–1.23)	0.49	1.10 (0.94–1.29)	0.25
	>70	1927	173 (8.98)	0.90 (0.74–1.08)	0.25	0.88 (0.73–1.07)	0.2	0.93 (0.77–1.14)	0.49

^a^ Adjusted for age; sex; height; body mass index; smoking; history of hypertension; history of diabetes; using statins at discharge; using antiplatelets at discharge; using anticoagulants at discharge; using antihypertensives at discharge.

^b^ Adjusted for age; sex; height; body mass index; smoking; history of hypertension, history of diabetes; using statins at discharge; using antiplatelets at discharge; using anticoagulants at discharge; using antihypertensives at discharge; baseline NIHSS score; white blood cells.

^c^ LVEF>60% as reference.

Abbreviations: CI, confidence intervals; HR, hazard ratios; LVEF, left ventricular ejection fraction; mRS, modified Rankin Scale; OR, odds ratios; Ref, reference.

The baseline characteristics and outcomes according to LVEF above and below 60% were further compared (Table S1). In a propensity‐matched analysis, LVEF ≤60% and >60% were matched on age, sex, height, BMI, history of ischemic stroke, baseline NIHSS score, toast classification. A total of 4153 matched pairs were identified. In multivariate logistic regression analyses, LVEF ≤60 was associated with higher risk of all‐cause death compared to LVEF >60% (aHR 1.29 [95% CI 1.06–1.58]; *p* = 0.01) after adjusting for all confounders considered in the study (Table S3).

### 3.3 Subgroup analyses

Results of subgroup analyses of the primary outcome are shown in Table 3. There was no heterogeneity in the effects of LVEF levels on the primary outcome between subgroups classified by age, sex, stroke cause, previous history of ischemic stroke, and baseline NIHSS score. Of note, patients with LVEF ≤60% were significantly associated with the primary outcome in certain subgroups of special interests, including male, patients with non‐cardiogenic stroke, baseline NIHSS score ≥4, and those without previous ischemic stroke, but there were no significant interactions.

**TABLE 3:** Subgroup analysis of primary outcome according to LVEF levels.

Outcomes	LVEF (%)	*N*	Events, *n* (%)	HR/OR (95% CI)	*p* Value	Adjusted HR/OR (95% CI)^a^	*p* Value	*p* for interaction
Age								
≤60 years	≤60	1609	30 (1.86)	1.59 (1.02–2.50)	0.04	1.42 (0.89–2.27)	0.14	0.76
	>60	4342	51 (1.17)	Ref.		Ref.		
>60 years	≤60	2593	138 (5.32)	1.49 (1.20–1.85)	<0.001	1.36 (1.09–1.70)	0.006	
	>60	5509	199 (3.61)	Ref.		Ref.		
Sex								
Male	≤60	2953	110 (3.73)	1.64 (1.28–2 10)	<0.001	1.33 (1.03–1.71)	0.03	0.80
	>60	6632	152 (2.29)	Ref.		Ref.		
Female	≤60	1249	58 (4.64)	1.54 (1.11–2.13)	0.009	1.21 (0.87–1.69)	0.26	
	>60	3219	98 (3.04)	Ref.		Ref.		
Stroke cause								
Cardiogenic	≤60	400	30 (7.50)	1.37 (0.81–2.34)	0.24	1.32 (0.76–2.28)	0.32	0.97
	>60	458	25 (5.46)	Ref.		Ref.		
Non‐Cardiogenic	≤60	3802	138 (3.63)	1.53 (1.24–1.89)	<0.001	1.28 (1.03–1.58)	0.03	
	>60	9393	225 (2.40)	Ref.		Ref.		
Previous ischemic stroke								
Yes	≤60	907	49 (5.40)	1.33 (0.94–1.90)	0.11	1.15 (0.80–1.65)	0.45	0.30
	>60	2005	82 (4.09)	Ref.		Ref.		
No	≤60	3295	119 (3.61)	1.70 (1.35–2.15)	<0.001	1.36 (1.07–1.74)	0.01	
	>60	7846	168 (2.14)	Ref.		Ref.		
Baseline NIHSS score								
≥4	≤60	2007	123 (6.13)	1.64 (1.30–2.08)	<0.001	1.39 (1.09–1.76)	0.008	0.31
	>60	4317	163 (3.78)	Ref.		Ref.		
0–3	≤60	2195	45 (2.05)	1.31 (0.91–1.88)	0.14	1.12 (0.78–1.62)	0.53	
	>60	5534	87 (1.57)	Ref.		Ref.		

Abbreviation: NIHSS, National Institutes of Health Stroke Scale.

^a^ Adjusted for age; sex; height; body mass index; smoking; history of hypertension, history of diabetes; using statins at discharge; using antiplatelets at discharge; using anticoagulants at discharge; using antihypertensives at discharge; baseline NIHSS score; white blood cells.

## 4 DISCUSSION

In this large, long‐term follow‐up, prospective registry across China, we investigated the association between routinely echocardiographic assessed LVEF spectrum with 5% width intervals and clinical outcomes in patients with acute ischemic cerebrovascular disease. We found that LVEF ≤60% was associated with a higher risk of all‐cause death compared to LVEF >60%, and the risk of all‐cause death increased gradually with progressive LVEF reduction. Our study added new data to this research area by accessing this association in the broad LVEF spectrum. These results highlight that LVEF 50–60%, although within the normal range, may still contribute to poor outcomes in acute ischemic cerebrovascular disease. Assessment and monitoring of cardiac function are also important in these populations.

Our current study showed that patients with acute ischemic cerebrovascular disease with lower LVEF were generally older, more likely to have a history of heart diseases, higher NHISS on admission, a higher rate of cardiogenic embolism in TOAST types, a higher percentage of receiving anticoagulant agents at discharge accordingly, which was in line with previous findings.^3^, ^4^, ^10^

The reported prevalence of decreased LVEF in stroke patients ranges from 5% to 20%,^3^, ^4^, ^5^, ^6^ while our registry studies have demonstrated that the number of patients with acute ischemic cerebrovascular disease with reduced LVEF (<50%) is less common (2.45%). Patients with LVEF <50% were reported to represent nearly 20% of the community population but included older patients and a higher proportion of patients with a history of heart disease.^2^

Recent research has shown that several factors can influence the prognosis of stroke including demographic characteristics,^11^ time to treatment,^12^, ^13^ comorbidities,^14^, ^15^ blood markers,^16^ imaging findings,^17^ and so on. Identifying the factors that influence stroke prognosis helps clinicians predict outcomes, guide treatment decisions, and inform the development of new interventions. Our study provides new insight to this area by finding the association between readily available clinical indicator LVEF and survival in AIS or TIA patients.

There are conflicting results regarding the association of cardiac function (with or without LVEF data) and outcomes, including functional disability and stroke recurrence in stroke patients.^3^, ^4^, ^5^, ^6^, ^18^, ^19^, ^20^ These inconsistent results may be partly due to the different definitions of heart failure and category of LVEF level. Our study focused on LVEF levels, which could be routinely obtained in clinical practice in patients with acute ischemic cerebrovascular disease. LVEF grouping methods employed in previous studies have only targeted specific ranges or used a crude dichotomization.^4^, ^21^ Our study assessed the association of a broad LVEF spectrum with clinical outcomes in a large, long‐term follow‐up, prospective registry. We found that in patients with acute ischemic cerebrovascular disease with LVEF ≤60%, LVEF levels independently predicted the 1‐year risk of all‐cause death, and the risk gradually increased with successive decreases in LVEF levels. Our study demonstrates that left ventricular systolic function is more closely related to mortality in patients with acute ischemic cerebrovascular disease, and even an LVEF of 50%–60%, which is generally considered to be normal, still has an increased risk of mortality. Therefore, comprehensive evaluation of cardiac function after AIS or TIA should be strengthened. Our study added new data to this research area by accessing this association in the broad LVEF spectrum.

The reasons underlying the associations between lower LVEF and poor outcomes in patients with AIS or TIA were unclear and may be multifactorial. The etiology of AIS patients with lower LVEF was more likely to be cardioembolism in the TOAST classification, which may account for the poorer prognosis in this population. Furthermore, given the close relationship between heart disease and LVEF levels, heart disease may be one of the contributing factors to this association. Patients with lower LVEF were more likely to receive anticoagulation treatment, which was associated with a higher risk of hemorrhagic complications leading to worse outcomes.^22^ In addition, identical conclusions that AIS patients with lower LVEF have poorer neurological function at admission were obtained in studies, which may be explained by autonomic dysfunction after vascular brain injury.^23^ Notably, an essential finding of this study was that patients with lower LVEF had higher levels of WBC and C‐reactive protein, which were commonly regarded as sensitive indicators of inflammation. It has been found that patients with lower LVEF suffered more severe inflammation burden,^24^, ^25^ but this has not been verified in stroke patients. Our findings suggest that ischemic stroke patients with decreased LVEF may experience a greater inflammatory burden than patients with normal LVEF. Growing evidence suggests that the immune system plays a complex role in the pathophysiological changes following cerebral ischemia injury.^26^ Subsequent neuroinflammatory and systemic inflammatory processes following ischemia induce neuronal dysfunction, leading to larger infarct volumes and worse clinical outcomes.^16^, ^26^ The underlying mechanisms of inflammatory response and poor outcomes in ischemic stroke patients with ventricular dysfunction require further study. In our study, after adjusting for several variables in multivariate analysis, including age, sex, BMI, baseline NIHSS score, WBC, treatment, and risk factors, the association between lower LVEF and 1‐year all‐cause death remained statistically significant, suggesting that there may be other potential factors at play. Our study demonstrates that LEVF ≤60% is an independent predictor of death in patients with AIS and highlights that values of 50%–60%, although within the borderline normal LVEF, may still contribute to poor outcomes in acute ischemic cerebrovascular disease.

Limitations of this study warrant discussion. First, although this largest sample size of the study revealed an association between LVEF values and stroke outcomes, the number of patients with reduced LVEF was relatively small. This may explain why mortality in the LVEF 56%–60% group did not reach statistical significance. Second, this study lacks of comprehensive evaluation of cardiac structure and function due to without advanced technologies such as three‐dimensional echocardiography and cardiac MRI. The advantage of two‐dimensional echocardiography is that it is a simple and reliable method for assessing cardiac structure and function, which is easily and widely available in clinical practice. Third, this study did not include an analysis of detailed treatment information, such as prescribed doses of antiplatelets, anticoagulants, or antihypertensive drugs, which may affect clinical prognosis due to different efficacy and safety. Fourth, the reasons underlying the associations between lower LVEF and poor outcomes in patients with AIS or TIA were unclear. Further studies are needed to clarify this issue.

## 5 CONCLUSION

Our study demonstrated that lower LVEF was associated with older age, cardiac comorbidities, more severe neurological deficits, and higher levels of inflammatory markers. LVEF ≤60% was associated with a higher risk of all‐cause death compared to LVEF >60%, and the risk of all‐cause death increased gradually with decreasing LVEF levels. LVEF values of 50–60%, although within the normal range, may still contribute to poor outcomes in acute ischemic cerebrovascular disease. Future research needs to explore its underlying mechanism.

## FUNDING INFORMATION

This study was funded by the National Natural Science Foundation of China (81,820,108,012, 82,071,301).

## CONFLICT OF INTEREST STATEMENT

None.

## Supporting information


Tables S1–S3

## Data Availability

Data related to the current study are available from the corresponding author on reasonable request.
