# Supplementary material for: Effect of left ventricular ejection fraction Spectrum on 1‐Year mortality in patients with acute ischemic stroke or transient ischemic attack
Source: CNS Neurosci Ther. 2023 Jun 7;29(11):3518–27. doi: 10.1111/cns.14285 (PMC10580366; doi:10.1111/cns.14285)
Supplement: Supplementary file 1 — Tables S1–S3 [file CNS-29-3518-s001.docx]

Table S1. Baseline Characteristics and Outcomes according to LVEF Levels.

| Characteristics | Total  (N=14,053) | LVEF≤60%  (n=4,202) | LVEF >60%  (n=9,851) | p Value |
| --- | --- | --- | --- | --- |
| Age (years), mean ±SD | 62.23±11.24 | 63.37±11.33 | 61.75±11.17 | <0.001^*^ |
| Male,n (%) | 9,585(68.21) | 2,953(70.28) | 6,632(67.32) | <0.001^*^ |
| Height(cm), mean ±SD | 166.87±7.47 | 167.43±7.46 | 166.63±7.46 | <0.001^*^ |
| BMI(kg/m^2^), mean ±SD | 24.72±3.34 | 24.84±3.43 | 24.66±3.30 | 0.005^*^ |
| Medical history |  |  |  |  |
| Ischemic stroke, n(%) | 2,912(20.72) | 907(21.58) | 2,005(20.35) | 0.10 |
| Hypertension, n(%) | 8,800(62.62) | 2,618(62.30) | 6,182(62.76) | 0.61 |
| Diabetes mellitus, n(%) | 3,229(22.98) | 975(23.20) | 2,254(22.88) | 0.68 |
| Dyslipidemia, n(%) | 1,117(7.95) | 324(7.71) | 793(8.05) | 0.50 |
| Chronic kidney disease, n(%) | 116(0.83) | 31(0.74) | 85(0.86) | 0.45 |
| Valvular heart defect, n(%) | 59(0.42) | 21(0.50) | 38(0.39) | 0.34 |
| Heart failure, n(%) | 90(0.64) | 60(1.43) | 30(0.30) | <0.001^*^ |
| Positive smoking history, n(%) | 4,407(31.36) | 1,273(30.30) | 3,134(31.81) | 0.08 |
| NIHSS at admission, median (IQR) | 3(1-6) | 3(1-6) | 3(1-6) | <0.001^*^ |
| Laboratory test |  |  |  |  |
| Hematocrit (%), median (IQR) | 41.10(36.90-44.50) | 42.00(39.00-45.00) | 42.00(39.10-45.00) | 0.39 |
| WBC (10^3^/mL), median (IQR) | 6.90(5.70-8.40) | 6.98(5.71-8.49) | 6.89(5.70-8.40) | 0.16 |
| C-reactive protein (mg/L), median (IQR) | 1.74(0.81-4.64) | 1.95(0.90-5.47) | 1.63(0.78-4.29) | <0.001^*^ |
| Total cholesterol (mmol/L), median (IQR) | 3.97(3.31-4.71) | 4.00(3.34-4.77) | 3.95(3.29-4.69) | 0.02^*^ |
| Triglyceride (mmol/L), median (IQR) | 1.37(1.03-1.87) | 1.34(1.02-1.82) | 1.39(1.04-1.90) | 0.001^*^ |
| HDL-C (mmol/L), median (IQR) | 0.93(0.77-1.12) | 0.95(0.78-1.13) | 0.93(0.77-1.11) | 0.001^*^ |
| LDL-C (mmol/L), median (IQR) | 2.31(1.72-2.97) | 2.36(1.76-3.03) | 2.28(1.70-2.95) | <0.001^*^ |
| Fasting blood glucose (mmol/L), median (IQR) | 5.51(4.89-6.87) | 5.54(4.90-7.04) | 5.50(4.89-6.80) | 0.10 |
| Glycated hemoglobin (%), median (IQR) | 5.90(5.50-6.90) | 6.00(5.50-7.00) | 5.90(5.50-6.90) | 0.04^*^ |
| Blood urea nitrogen (mmol/L), median (IQR) | 4.95(4.08-6.07) | 5.10(4.13-6.30) | 4.88(4.00-5.91) | <0.001^*^ |
| Creatinine (umol/L), median (IQR) | 69.00(59.00-82.00) | 71.00(60.00-84.00) | 69.00(58.00-80.00) | <0.001^*^ |
| Medication at discharge, n (%) |  |  |  |  |
| Statins | 12,852(91.45) | 3,770(89.72) | 9,082(92.19) | <0.001^*^ |
| Antihypertensives | 6,948(49.44) | 2,069(49.24) | 4,879(49.53) | 0.75 |
| CCB | 5,200(37.00) | 1,504(35.79) | 3,696(37.52) | 0.052 |
| ACEI | 688(4.90) | 226(5.38) | 462(4.69) | 0.08 |
| ARB | 1,839(13.09) | 556(13.23) | 1,283(13.02) | 0.74 |
| DIU | 325(2.31) | 124(2.95) | 201(2.04) | 0.001^*^ |
| beta-receptor blockers | 595(4.23) | 243(5.78) | 352(3.57) | <0.001^*^ |
| alpha-receptor blockers | 14(0.10) | 3(0.07) | 11(0.11) | 0.69 |
| Others | 197(1.40) | 70(1.67) | 127(1.29) | 0.08 |
| Antiplatelets | 12,764(90.83) | 3,669(87.32) | 9,095(92.33) | <0.001^*^ |
| Mono antiplatelet | 8,473(60.29) | 2,466(58.69) | 6,007(60.98) | <0.001^*^ |
| Dual antiplatelet | 4,291(30.53) | 1,203(28.63) | 3,088(31.35) |  |
| None | 1,289(9.17) | 533(12.68) | 756(7.67) |  |
| Anticoagulants | 420(2.99) | 198(4.71) | 222(2.25) | <0.001^*^ |
| Outcomes at 3 months, n(%) |  |  |  |  |
| Functional dependence | 1,680(12.24) | 523(12.83) | 1,157(11.99) | 0.17 |
| Mortality | 169(1.20) | 75(1.78) | 94(0.95) | <0.001^*^ |
| Outcomes at 1 year, n(%) |  |  |  |  |
| Functional dependence | 1,348(10.12) | 426(10.83) | 922(9.83) | 0.08 |
| Mortality | 418(2.97) | 168(4.00) | 250(2.54) | <0.001^*^ |

Abbreviations: LVEF, left ventricular ejection fraction; SD, standard deviation; BMI, body mass index; NIHSS, National Institutes of Health Stroke Scale; IQR, interquartile range; WBC, white blood cells; HDL-C, high-density lipoprotein cholesterol; LDL-C, low-density lipoprotein cholesterol; CCB, calcium channel blockers; ACEI, angiotensin-converting enzyme inhibitors; ARB, angiotensin-receptor blockers; DIU, diuretic.

^*^p < 0.05.

Table S2. Association of LVEF With Clinical Outcome in Patients Without Heart Diseases

| Outcomes | LVEF (%) | Patients without heart diseases  (n=12068) | | | | |
| --- | --- | --- | --- | --- | --- | --- |
|  |  | Events, n (%) | Crude HR/OR (95% CI) | p Value | Adjusted HR/OR (95% CI) ^†^ | p Value |
| All-cause death |  |  |  |  |  |  |
|  | 0-40 | 5(11.11) | 5.02(2.04-12.35) | <0.001 | 3.99(1.62-9.86) | 0.003 |
|  | 41-45 | 1(2.86) | 1.27(0.18-9.08) | 0.81 | 0.99(0.14-7.14) | 0.99 |
|  | 46-50 | 7(7.95) | 3.42(1.58-7.37) | 0.002 | 2.06(0.89-4.74) | 0.09 |
|  | 51-55 | 21(4.28) | 1.81(1.13-2.91) | 0.01 | 1.60(0.99-2.58) | 0.05 |
|  | 56-60 | 67(2.48) | 1 04(0 76-1 43) | 0.8 | 0.89(0.64-1.23) | 0.47 |
|  | 61-65 | 91(2.38) | ref. |  | ref. | -- |
|  | 66-70 | 59(1.93) | 0.81(0.58-1.12) | 0.2 | 0.84(0.60-1.17) | 0.29 |
|  | >70 | 42(2.30) | 0.96(0.67-1.39) | 0.85 | 0.97(0.67-1.40) | 0.87 |
| Stroke recurrence |  |  |  |  |  |  |
|  | 0-40 | 7(15.56) | 1.87(0.89-3.95) | 0.1 | 1.73(0.82-3.66) | 0.15 |
|  | 41-45 | 0 |  |  |  |  |
|  | 46-50 | 11(12.50) | 1.42(0.78-2.58) | 0.26 | 1.19(0.63-2.23) | 0.6 |
|  | 51-55 | 39(7.94) | 0.88(0.63-1.23) | 0.46 | 0.83(0.59-1.16) | 0.27 |
|  | 56-60 | 284(10.51) | 1.18(1.01-1.38) | 0.04 | 1.14(0.97-1.33) | 0.11 |
|  | 61-65 | 342(8.95) | ref. |  | ref. |  |
|  | 66-70 | 256(8.37) | 0.93(0.79-1.09) | 0.36 | 0.94(0.80-1.10) | 0.44 |
|  | >70 | 165(9.04) | 1.00(0.83-1.21) | 0.96 | 1.01(0.84-1.22) | 0.88 |
| mRS 3-5 |  |  |  |  |  |  |
|  | 0-40 | 5(12.82) | 1.43(0.55-3.67) | 0.46 | 1.07(0.39-2.92) | 0.90 |
|  | 41-45 | 4(13.33) | 1.49(0.52-4.30) | 0.46 | 1.50(0.50-4.51) | 0.47 |
|  | 46-50 | 10(12.50) | 1.39(0.71-2.71) | 0.34 | 1.16(0.58-2.35) | 0.67 |
|  | 51-55 | 57(12.31) | 1.36(1.01-1.84) | 0.04 | 1.18(0.86-1.61) | 0.32 |
|  | 56-60 | 238(9.26) | 0.99(0.83-1.18) | 0.91 | 0.95(0.79-1.14) | 0.57 |
|  | 61-65 | 341(9.35) | ref. |  | ref. |  |
|  | 66-70 | 280(9.54) | 1.02(0.87-1.21) | 0.79 | 1.06(0.89-1.26) | 0.55 |
|  | >70 | 155(8.89) | 0.95(0.78-1.15) | 0.58 | 0.96(0.78-1.18) | 0.69 |

† Adjusted for age; sex; height; body mass index; smoking; history of hypertension, history of diabetes; Using statins at discharge; Using antiplatelets at discharge; Using Anticoagulants at discharge; Using Antihypertensives at discharge; baseline NIHSS score; white blood cells

Table S3. Associations of LVEF With Clinical Outcomes at 1 Year in Propensity-Matched Analysis

| Outcomes | LVEF (%) | N | Events, n(%) | Crude HR/OR (95% CI) | p Value | Model 1  Adjusted HR/OR (95% CI) ^†^ | p Value | Model 2  Adjusted HR/OR (95% CI) ^‡^ | p Value |
| --- | --- | --- | --- | --- | --- | --- | --- | --- | --- |
| **Primary outcome** | | | | | | | | | |
| All-cause death |  |  |  |  |  |  |  |  |  |
|  | ≤60 | 4153 | 162(3.90) | 1.47(1.16-1.87) | 0.002 | 1.35(1.11-1.64) | 0.003 | 1.29(1.06-1.58) | 0.01 |
|  | >60 | 4153 | 111(2.67) | ref. |  | ref. |  | ref. |  |
| **Secondary outcome** | | | | | | | | | |
| Stroke recurrence |  |  |  |  |  |  |  |  |  |
|  | ≤60 | 4153 | 438(10.55) | 1.14(0.99-1.30) | 0.07 | 1.13(1.01-1.27) | 0.04 | 1.11(0.99-1.24) | 0.09 |
|  | >60 | 4153 | 389(9.37) | ref. |  | ref. |  | ref. |  |
| mRS 3-5 |  |  |  |  |  |  |  |  |  |
|  | ≤60 | 3889 | 412(10.59) | 0.91(0.79-1.05) | 0.20 | 0.90(0.78-1.04) | 0.14 | 0.91(0.79-1.06) | 0.24 |
|  | >60 | 3939 | 453(11.50) | ref. |  | ref. |  | ref. |  |

†Adjusted for age; sex; height; body mass index; smoking; history of hypertension, history of diabetes; Using statins at discharge; Using antiplatelets at discharge; Using Anticoagulants at discharge; Using Antihypertensives at discharge.

‡Adjusted for age; sex; height; body mass index; smoking; history of hypertension, history of diabetes; Using statins at discharge; Using antiplatelets at discharge; Using Anticoagulants at discharge; Using Antihypertensives at discharge; baseline NIHSS score; white blood cells.
